# A Cross-Species Gene Expression Marker-Based Genetic Map and QTL Analysis in Bambara Groundnut

**DOI:** 10.3390/genes8020084

**Published:** 2017-02-22

**Authors:** Hui Hui Chai, Wai Kuan Ho, Neil Graham, Sean May, Festo Massawe, Sean Mayes

**Affiliations:** 1 Biotechnology Research Centre, School of Biosciences, University of Nottingham Malaysia Campus, Jalan Broga, Semenyih 43500, Selangor Darul Ehsan, Malaysia; waikuan@cffresearch.org (W.K.H.); Festo.Massawe@nottingham.edu.my (F.M.); sean.mayes@cffresearch.org (S.M.); 2 Crops For the Future, Jalan Broga, Semenyih 43500, Selangor Darul Ehsan, Malaysia; 3 Plant and Crop Sciences, School of Biosciences, University of Nottingham, Sutton Bonington Campus, Leics, Loughborough LE12 5RD, UK; neil.graham@nottingham.ac.uk (N.G.); sean.may@nottingham.ac.uk (S.M.)

**Keywords:** Affymetrix GeneChip, cross-hybridisation, gene expression markers, quantitative trait loci

## Abstract

Bambara groundnut (*Vigna subterranea* (L.) Verdc.) is an underutilised legume crop, which has long been recognised as a protein-rich and drought-tolerant crop, used extensively in Sub-Saharan Africa. The aim of the study was to identify quantitative trait loci (QTL) involved in agronomic and drought-related traits using an expression marker-based genetic map based on major crop resources developed in soybean. The gene expression markers (GEMs) were generated at the (unmasked) probe-pair level after cross-hybridisation of bambara groundnut leaf RNA to the Affymetrix Soybean Genome GeneChip. A total of 753 markers grouped at an LOD (Logarithm of odds) of three, with 527 markers mapped into linkage groups. From this initial map, a spaced expression marker-based genetic map consisting of 13 linkage groups containing 218 GEMs, spanning 982.7 cM (centimorgan) of the bambara groundnut genome, was developed. Of the QTL detected, 46% were detected in both control and drought treatment populations, suggesting that they are the result of intrinsic trait differences between the parental lines used to construct the cross, with 31% detected in only one of the conditions. The present GEM map in bambara groundnut provides one technically feasible route for the translation of information and resources from major and model plant species to underutilised and resource-poor crops.

## 1. Introduction

Bambara groundnut (*Vigna subterranea* (L.) Verdc) is an indigenous legume crop grown mainly by subsistence and small-scale farmers in Sub-Saharan Africa. This legume species is the third most important legume after groundnut (*Arachis hypogaea*) and cowpea (*Vigna unguiculata*) in semi-arid Africa [1]. In addition to relatively high protein content in bambara groundnut seed (18%–26%) [2,3], bambara groundnut is also recognised for its good drought tolerance and ability to grow in poor soils, the latter partly as a result of fixing nitrogen from the atmosphere.

Being a relatively neglected crop species, the cultivation of bambara groundnut has mainly relied on local experience and indigenous knowledge [4]. There are few established varieties of bambara groundnut, and most of the bambara groundnut accessions exist in the form of landraces [5]. Analysis using co-dominant markers [6] revealed that bambara groundnut is highly inbred, with seed from a single plant essentially representing an inbred line, although relatively high levels of genetic variation were observed between plants from the same landrace accession. Screening landraces could provide breeders with genetic resources to improve yield, biotic and abiotic resistance and the adaptability of the crop to various environments.

To understand the genetic architecture of bambara groundnut for breeding purposes, it is useful to develop a high density genetic linkage map. A genetic map facilitates the identification of important gene-containing regions prior to positional cloning or the use of flanking markers for marker-assisted selection (MAS) in breeding programmes. Genetic maps can assist with conserved synteny mapping, where markers can be located in the species of origin and comparisons made with the species where greater resources and trait knowledge exist. The first genetic map in bambara groundnut (2*n* = 2*x* = 22) was reported by Basu et al. [7] using an F_2_ segregating population derived from a controlled cross between an ancestral wild-type (VSSP11) and the domesticated form (a genotype derived from the DipC landrace) of bambara groundnut on the basis of differences observed in growth habit, maturity and yield performance. Twenty linkage groups were identified using 67 AFLP (Amplified Fragment Length Polymorphism) markers and one SSR (Simple Sequence Repeat) marker, giving a total length of 516 cM (centimorgan), with the inter-marker distance varying from 4.7 cM to 32 cM [7]. A quantitative trait loci (QTL) analysis localised four QTL controlling 100-seed weight, specific leaf area, number of stem per plant and carbon (Delta C^13^) isotope discrimination through the use of this interspecific genetic map [8]. In addition, Ahmad et al. (2016) [9] reported the construction of the first intraspecific genetic map using an F_3_ segregating population derived from a cross between genotypes derived from two domesticated landraces, Tiga Nicuru and DipC. The intraspecific map covered 608.3 cM over 21 linkage groups and included 29 SSR and 209 microarray-based DArT markers, with marker-marker distances ranging between 0 cM and 10.1 cM. QTL analysis allowed the mapping of 36 significant QTL for 19 agronomic traits, including internode length, peduncle length and biomass dry weight [9].

Construction of genetic maps has employed various types of DNA-based markers, while gene-based microarrays have been widely adopted in recent years to generate large-scale expression data. Recently, microarrays have been increasingly used to generate expression-based marker information, such as gene expression markers (GEMs), for the development of an expression-based genetic map, followed by comprehensive conventional quantitative trait locus (QTL) and expression quantitative trait loci (eQTL) studies [10,11,12,13,14]. GEMs refer to significant differences in the hybridisation signal strength between individuals when cRNA is hybridised to GeneChip arrays. The differences in hybridization strength observed between individuals could be due to either sequence polymorphisms effecting the hybridization efficiencies of nucleic acids to individual features on the microarrays or actual differences in the transcript (mRNA) abundance. In a structured population, such as a series of recombinant inbred lines (RILs) derived from a controlled cross, these differences can be interpreted as genetic markers for map construction. In one *Brassica* eQTL experiment, the majority of GEMs were selected based on differences in hybridisation signal intensity in the parental plants. These were likely to have resulted from (but were not unequivocally proven to be) sequence polymorphisms, which effected the binding of the test RNA samples to the microarray targets [14]. Using a genetic map constructed from 125 GEMs, Hammond et al. (2011) [14] showed that the identification of regulatory hotspots that regulated low phosphorus response in *B. rapa* was possible.

The potential for analysing less intensively studied species (such as bambara groundnut) using GEMs derived from a microarray was demonstrated when Hammond et al. (2005) [15] reported the hybridisation of RNA derived from *Brassica oleracea* L. plants responding to mineral nutrient (phosphorus; P) stress on the Affymetrix GeneChip designed for *Arabidopsis*, followed by the identification of 99 genes that were significantly different between parental samples under P stress in *B. oleracea*. The approach adopted, which uses microarrays developed for a given species to analyse another related species, is known as the XSpecies (cross-species) microarray approach (http://affy.arabidopsis.info/xspecies/). Affymetrix GeneChip microarrays can provide reproducible and accurate data, which can be compared across experiments. While custom genotyping arrays for species with genome sequence information could be designed despite the high manufacturing costs for a microarray, thirteen Affymetrix GeneChip microarrays are currently publicly available for limited numbers of plant species [16]. The XSpecies microarray approach, which is one of the approaches developed for the Affymetrix GeneChip platform, offers a potential approach to research crop species where no species-specific microarray is publically available. In addition to *Brassica*, the XSpecies microarray approach has been used in crop species, such as wheat (*Triticum aestivum*) [17], cowpea [18], banana (*Musa*) [19], sorghum (*Sorghum bicolor*) [20] and blueberry (*Vaccinium corymbosum*) [21].

The application of QTL analysis can lead to the identification of candidate genes that control the observed traits. For instance, a QTL for beginning of flowering in pea was mapped onto linkage group LGV at 49 cM [22], which harbours the gene, *DET*, that is involved in the regulation of flowering time and inflorescence architecture [23]. Using extensive genomic resources developed in major crops or closely-related species, conserved syntenic loci and potential candidate genes that control important agronomic traits in bambara groundnut could be detected by projecting the map of QTL onto a physical map or genetic map based on functional markers derived from closely-related species, such as soybean (*Glycine max*) and common bean (*Phaseolus vulgaris*).

The aim of this study was to identify the locations of quantitative trait loci for a wide range of agronomic traits displayed in a bambara groundnut F_5_ segregating population using the XSpecies approach, with microarray resources developed in soybean. Following cross-hybridisation of RNA from bambara groundnut to the Affymetrix Soybean Genome GeneChip, an expression-based genetic map (GEM map) was constructed and utilised for a comprehensive QTL analysis. The present GEM map will serve as a platform for conserved synteny mapping with closely-related crop species, such as soybean and common bean, for possible transfer of positional information, creation of conserved synthetic links and identification of candidate genes and gene regions for breeding.

## 2. Materials and Methods

### 2.1. Plant Material

An F_5_ segregating population was derived from a controlled cross between single genotypes derived from the Tiga Nicuru (maternal) and DipC (paternal) landraces. The irrigated population received a continuous watering regime, while the drought treatment population was subjected to a 42-day cumulative mild drought treatment starting at 50 DAS (days after sowing) by withholding irrigation prior to genetic linkage analysis. Plant material, consisting of parental lines and 65 F_5_ individual lines, was planted in a controlled environment glasshouse at the FutureCrop Glasshouses, Sutton Bonington Campus, the University of Nottingham, U.K. The details of the plant material and experimental design were reported in Chai et al. (2016) [24].

### 2.2. RNA Preparation and Cross-Hybridisation

After bambara groundnut parental lines and the drought treatment F_5_ segregating population had received six weeks of drought treatment, two leaves from each of the individual plants were harvested. In total, 77 biological samples were collected, comprising one biological replicate for each line from the drought treatment plot (*n* = 65) and three replicates of each parental line under drought and irrigation conditions (3 replicates × 2 parents × 2 treatments = 12). The leaf samples were then transferred to a −80 °C freezer for longer term storage. Total RNA was extracted using the Qiagen RNeasy Plant Mini Kit (Qiagen, Manchester, U.K.) according to the manufacturer’s instructions. The final RNA was resuspended in 30 μL RNase-free water. Total RNA was checked for integrity and quality using both the Agilent 2100 Bioanalyzer (Agilent Technologies, Santa Clara, CA, USA) and gel electrophoresis. A total of 10 μL of RNA samples (100 ng·μL^−1^) derived from the 12 parental samples and 65 individual lines were sent to the NASC Affymetrix Service, UoN, Sutton Bonington Campus, U.K., for cross-hybridisation analysis onto the Affymetrix Soybean Genome GeneChip.

### 2.3. Generation of GEMs

A total of 77 data files was generated and initial data analysis performed using GeneSpring GX (Version 11.0.2; Agilent Technologies). The analysis approach adopted for bambara groundnut data was based on, but modified from, Hammond et al. (2011) [14], which used *B. rapa* as the experimental organism. Three sets of normalised data were produced at three different levels: probe-sets, chip definition file (CDF) masked probe-sets and unmasked probe-pairs (oligonucleotide). A new custom CDF file was created using PIGEONSv1.2 software [25] by filtering the original Tiga Nicuru CDF file and DipC CDF file at a signal threshold of 141. The custom CDF file was then used to mask the signals derived from each probe-set in order to generate a custom masked probe-set dataset. The three sets of normalised chip data were used to generate potential GEMs. The mean and standard deviation (s.d.) of each log2-normalised hybridisation signal were calculated for each of the parents and segregating population, followed by provisionally assigning allele scores “a” and “b” to each individual line for each putative marker, depending on if the hybridisation signal of individual line was on the same side of the mean population signal as the maternal (Tiga Nicuru) or parental (DipC) values (Supplemental Text S1). A good separation between group “a” and group “b” was indicated by high “distinctness” score. The probe-sets or probe-pairs in their respective normalised datasets with distinctness score equal or higher than a selected threshold value were selected as potential GEMs. The threshold values of markers used in map construction were retrospectively checked through visual inspection of the graphical distribution of group “a” and group “b”. A good separation of “a” and “b” allele scores within the individual lines would allow the production of polymorphic GEMs of good quality.

### 2.4. Development of Expression-Based Linkage Map Using GEMs

The GEM map was constructed using JoinMap v4.1 [26]. A combination of regression mapping (RM) and maximum likelihood mapping (MLM) approaches with grouping at LOD ≥ 3 was applied to obtain the GEM order for each linkage group. The Haldane mapping function was used with default settings: recombination fraction ≤4.0, ripple value = 1, jump in goodness-of-fit threshold = 5. The order of GEMs was tested by comparing the results from RM and MLM. Miscalled GEMs showing double recombination events in individual lines within genetic distances between 1 and 3 cM were removed. The reiterative process of marker removal on the basis of visual inspection of graphical genotypes and by examining “fit and stress” parameters enables a high quality spaced framework map consisting of GEMs to be generated for further development and used as the basis for QTL analysis. Due to the insufficient linkage to complete the map using RM for linkage groups LG6 and LG8, these groups were divided into subgroups ”A” and “B”, respectively. However, MLM indicated that they were actually part of the same linkage groups.

### 2.5. Identifying Significant QTL

Data from a range of morphological and physiological traits, including days to emergence, days to flowering, estimated days to podding, internode length, peduncle length, pod number per plant, pod weight per plant, seed number per plant, seed weight per plant, 100-seed weight, shoot dry weight and harvest index, and drought-related traits, including stomatal conductance, relative water content, stomatal density, leaf carbon (Delta C^13^) isotope analysis (CID) and leaf (Delta N^15^) isotope analysis (NID), were checked for normality and transformed, if necessary, as reported in Chai et al. (2016) [24] before being subjected to QTL analysis. MapQTL^®^ v6.1 (Kyazma, Wageningen, Netherlands) with a combination of two mapping approaches, interval mapping (IM) and multiple QTL mapping (MQM), was used to identify the QTL. The analysis options were set as default, including using the regression algorithm for IM and MQM mapping and fitting dominance for the population types. The permutation test using 10,000 reiterations was first conducted in order to determine the expected significance threshold of the LOD score, followed by IM mapping. Significant QTL were identified if the LOD score obtained from IM mapping was equivalent or higher than the genome-wide (GW) threshold at *p* ≤ 0.05 as derived from the permutation test. QTL were considered as ‘putative’ when the LOD score was lower than the GW threshold by up to one LOD. Once QTL with significant LOD scores were identified from the IM mapping model, the closest linked marker was selected as a cofactor prior to MQM mapping. If the result from MQM mapping showed a significant LOD score some distance away (a minimum of 20 cM) from the marker cofactor, automated cofactor selection (ACS) was applied for cofactor selection and MQM mapping repeated. The positions of QTL picked up by marker cofactors were verified through visual inspection of the LOD profile and the LOD table produced by MapQTL^®^ v6.1.

## 3. Results

### 3.1. Novel Gene Expression Markers

**Figure 1:**
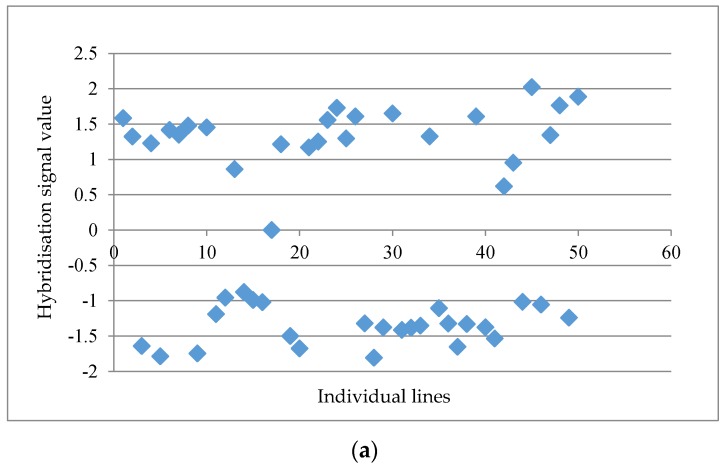

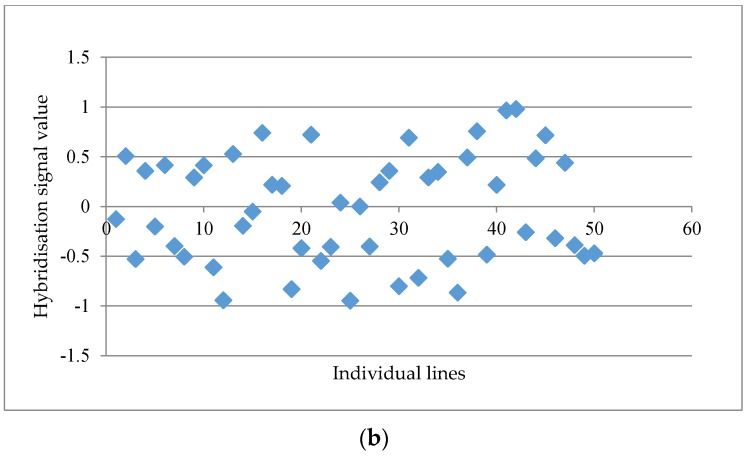
Examples of the ‘expression trait’ distribution of ‘a’ and ‘b’ allele scores across the individual lines at the unmasked probe-set level. (**a**) The probe set ‘GmaAffx.92555.1.S1_s_at’ with a distinctness score of 4.03 and (**b**) probe set ‘GmaAffx.57563.1.S1_at’ with a distinctness score of 2.15. The hybridisation signal value was normalised using GeneSpring GX. Allele ‘a’ (above the mid-point of the hybridisation scale axis, in these cases) was inherited from Tiga Nicuru, while allele ‘b’ was from DipC. Note the data point in (a) at the mid-point, which was scored as missing data, but could represent a heterozygote.

Three rounds of analysis on subsets of normalised data at different levels, probe-sets, CDF masked probe-sets and unmasked probe-pairs (oligonucleotide), were conducted (Table 1). The post analyses on a data matrix with 61,035 probe-sets at the probe-set level showed a maximum distinctness score of 4.03 followed by 3.44, 3.14 and 3.05 for the top four markers, whereas the lowest distinctness score was 1.18. A threshold value of 2.50 was set in order to obtain a relatively good separation between “a” and “b” alleles across the individual lines, as indicated by visual inspection. The probe set ‘GmaAffx.92555.1.S1_s_at’ with a distinctness score of 4.03 is presented in Figure 1a. When the distinctness score fell below 2.50, a more scattered graph was usually observed, such as the probe set “GmaAffx.57563.1.S1_at” with a distinctness score of 2.15 (Figure 1b). A total of 15 potential GEMs with a distinctness score of ≥2.50 were retrieved from the data matrix based on unmasked probe-sets in the first round of analysis.

The second round analysis retained a total number of 53,651 CDF masked probe-sets after filtering based on a genomic DNA hybridisation using an average of both of the parental lines, giving a threshold of 141. The post-analysis results gave a higher distinctness score of up to 6.09, followed by 5.23 and 5.21 for the top three markers after application of the CDF mask, compared to a maximum distinctness score of 4.03 in the previous analysis. Based on a visual inspection of the graphical distribution of “a” and “b” allele scores across the individual lines, a minimum distinctness score of 2.60 for the CDF masked dataset was adopted in order to obtain reasonably clear separation. In total, there were 48 potential GEMs with distinctness score of ≥2.60 extracted from the CDF masked dataset.

Although the number of potential GEMs generated from CDF masked probe-sets had improved, the relatively low number of GEMs retained was still insufficient to generate a comprehensive linkage map. Normalised data containing unmasked probe-pairs were used in the third round of analysis. The hybridisation signal values of the individual unmasked probe-pairs for each probe-set were calculated after chip normalisation, from 669,982 perfect-match probes (PM) and 669,982 mismatch probes (MM), respectively. For PM probes, the result showed that the highest distinctness score was 7.99 (Gma.3025.1.S1_at; PM-933459), followed by 7.30 (GmaAffx.69054.1.S1_s_at; PM-460879) and 5.31 (Gma.15877.1.S1_at; PM-41730) for the top three markers. A distinctness score of 2.34 was set as a cut-off point for good separation between “a” and “b” alleles across individual lines, resulting in a total number of 1029 potential GEMs. MM probes also presented a similar result in which a distinctness score of up to 7.10 was obtained by Gma.289.1.S1_s_at; MM-1048658, followed by 4.73 (Gma.17784.1.S1_at; MM-177722) and 4.47 (Gma.15877.1.S1_at; MM- 42894) for the top three markers. As a result, 501 potential GEMs were generated from MM probes where a threshold value of 2.30 was selected based on the graphical distribution of group “a” and “b” across all individual lines.

### 3.2. Development of an Expression-Based Genetic Map

Potential GEMs that showed an extreme distribution of alleles within the population were eliminated from the list. Of 1530 markers (1029 from PM probes and 501 from MM probes), 753 potential GEMs were identified and subjected to linkage analysis. The segregation pattern of the GEMs was examined using a chi-square test against the expected patterns. The result showed that only 55 markers (7.3%) presented significant (*p* < 0.1) segregation distortion, whereas 698 GEMs segregated in a way consistent with the expected Mendelian ratio for an F_5_ segregating population.

**Figure 2:**
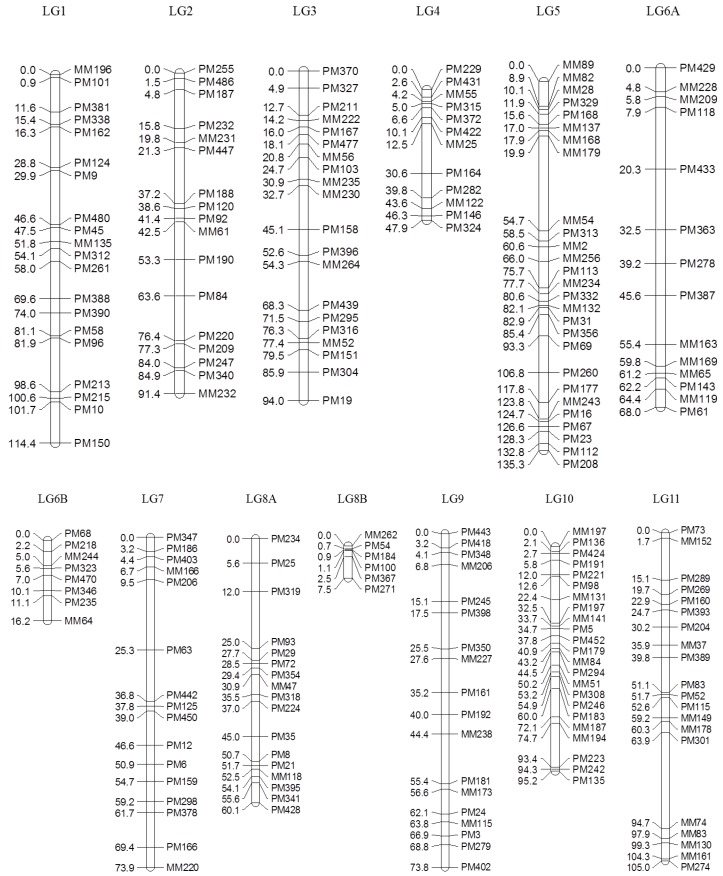
Genetic linkage map of the F_5_ segregating population in bambara groundnut constructed using gene expression markers (GEMs). Right: positions of markers (cM); left: name of the markers. LG6 and LG8 were divided into subgroups ‘A’ and ‘B’, respectively, based on the association observed in the MLM due to insufficient linkage to complete the map using regression mapping (RM).

Of 753 GEMs, 527 markers were provisionally mapped before removal of markers during the construction of a spaced framework map for QTL analysis. The spaced marker linkage analysis at LOD ≥ 3 generated 13 linkage groups with 218 GEMs (159 PM probes and 59 MM probes), spanning 982.7 cM of the bambara groundnut genome based on the regression mapping approach implemented in JoinMap4.1 (Table 2). There was an average of 16.8 markers per linkage group with the highest number (27) observed in LG5, followed by LG10 (23). Overall, the average distance between two neighbouring markers across all linkage groups was 4.2 cM, after the spaced map was created. The longest interval between markers was 34.8 cM in LG5 between MM179 (19.9 cM) and MM54 (54.7 cM). An average map length of 75.6 cM was calculated across all 13 linkage groups. LG5 appeared to be the longest linkage group and covered 135.3 cM with 27 GEMs, followed by LG1 (114.4 cM) and LG11 (105.0 cM; Figure 2).

### 3.3. Comparison of the Irrigated and Drought Treatment Population for QTL Associated with Agronomic Traits

**Figure 3:**
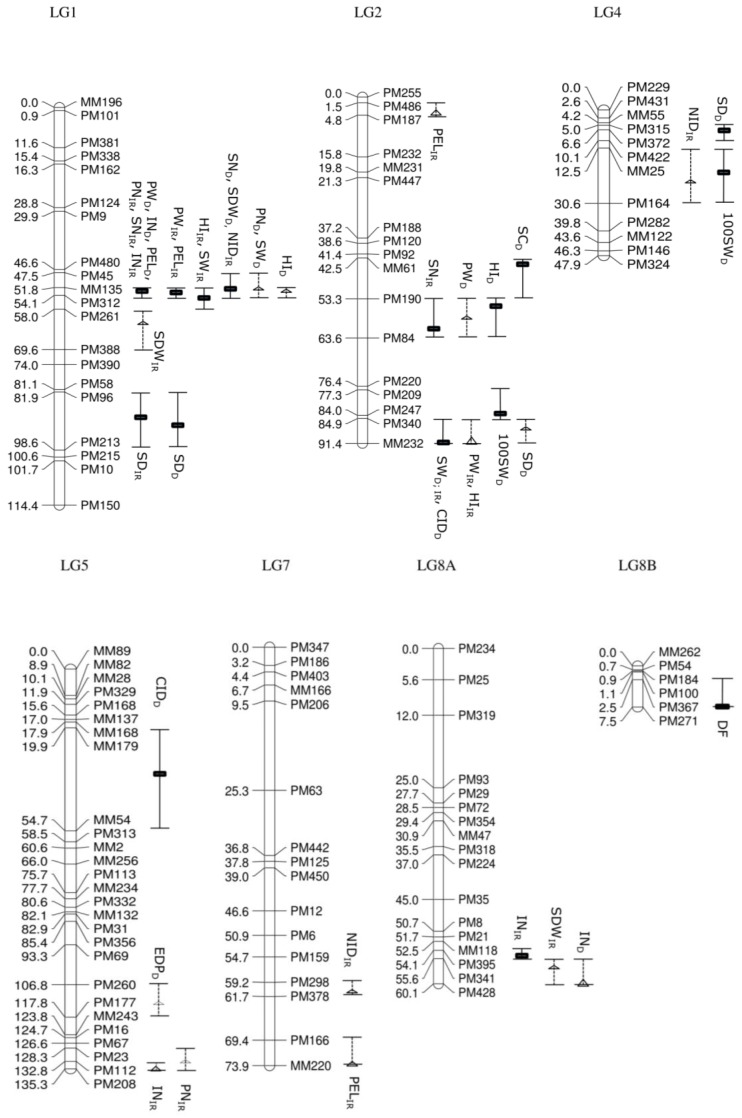

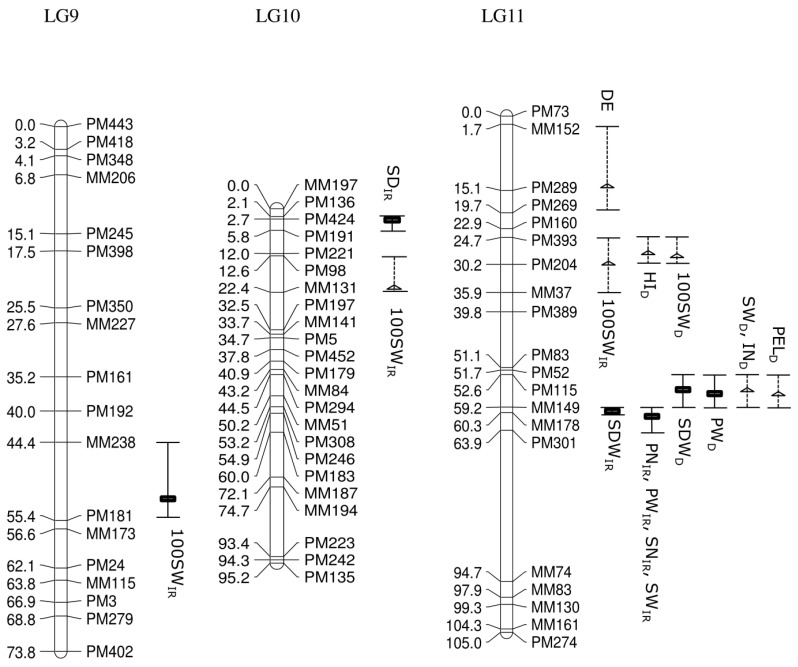
Map positions of the quantitative trait loci (QTL) in the irrigated (control) and drought treatment F_5_ segregating population developed from a cross between Tiga Nicuru and DipC. GEMs’ identity is described on the right and map positions (cM) on the left. The rectangular box (▬) with the solid confidence intervals indicates the location of significant QTL and their flanking markers, whereas the triangular box (Δ) with dotted confidence intervals represents putative QTL and their neighbouring markers. DE, days to emergence; DF, days to flowering; EDP, estimated days to podding; IN, internode length; PEL, peduncle length; PN, pod number per plant; PW, pod weight per plant; SN, seed number per plant; SW, seed weight per plant; 100SW, 100-seed weight; SDW: shoot dry weight; HI, harvest index; SC, stomatal conductance; CID, leaf carbon (Delta C^13^) isotope analysis; SD, stomatal density; NID, leaf (Delta N^15^) isotope analysis.

Days to emergence and days to flowering, which were recorded in the segregating population before imposing drought treatment, showed a putative QTL (15.3%; LOD_p_ = 2.12) on LG11 and a significant QTL (25.6%; LOD_s_ = 3.79) on LG8B, respectively. A total of 26 QTL (16 significant QTL and 10 putative QTL) associated with nine studied traits, distributed over eight linkage groups, was produced using IM and MQM in the irrigated population. Estimated days to podding had no significant QTL, which mapped onto the GEM genetic linkage map. In comparison, the drought treatment population produced 22 QTL associated with 10 agronomic traits distributed over six linkage groups with 11 significant QTL and 11 putative QTL (Figure 3).

Most of the QTL were clustered, especially on LG1, LG2 and LG11 in the irrigated population. Of 26 QTL, eight QTL (seven significant QTL and one putative QTL) were located on LG1, whereas five other QTL were identified on LG2 (two significant QTL and three putative QTL) and six QTL on LG11 (five significant QTL and one putative QTL). The clusters of QTL in the drought treatment population were also focused on three linkage groups, with eight QTL on LG1 (five significant QTL and three putative QTL), four QTL on LG2 (three significant QTL and one putative QTL) and seven QTL on LG11 (two significant QTL and five putative QTL).

A number of traits are likely to be correlated (Table S1). Some of the QTL have overlapping confidence intervals, opening up the possibility that they are being influenced by the same underlying gene. For example, QTL controlling seven traits—internode length, peduncle length, pod number per plant, seed number per plant, pod weight per plant, seed weight per plant and harvest index—in the irrigated population were detected at loci closely linked with MM135 (51.8 cM) and PM312 (54.1 cM) on LG1. Specifically, QTL for internode length (42.9%; LOD_s_ = 8.72), pod number per plant (33.9%; LOD_s_ = 7.01) and seed number per plant (28.1%; LOD_s_ = 6.47) were identified at 52.8 cM, QTL for peduncle length (41.1%; LOD_s_ = 8.93) and pod weight per plant (18.9%; LOD_s_ = 4.57) at 53.8 cM and QTL for seed weight per plant (13.4%; LOD_s_ = 3.25) and harvest index (24.5%; LOD_s_ = 5.43) at 54.1 cM, although the confidence intervals are all overlapping. The potentially pleiotropic effect of genes underlying the QTL could be further observed in QTL located on LG11 at loci closely linked with marker MM178 (60.3 cM), such as QTL controlling pod number per plant (13.5%; LOD_s_ = 3.15), seed number per plant (15.7%; LOD_s_ = 3.82), pod weight per plant (15.1%; LOD_s_ = 3.57) and seed weight per plant (16.5%; LOD_s_ = 3.99). In addition, the cluster of QTL located at 91.4 cM with linked marker MM232 on LG2 consisted of one significant QTL controlling seed weight per plant (13.6%; LOD_s_ = 3.39) and one putative QTL controlling pod weight per plant (10.4%; LOD_p_ = 2.56) and harvest index (11.1%; LOD_p_ = 2.73).

Some traits were shown to be controlled by multiple loci across different linkage groups. For example, internode length in the irrigated population had significant QTL, which mapped at loci on LG1 and LG8A, which has closely linked markers MM135 (51.8 cM) and PM341 (55.6 cM), respectively, and one putative QTL with marker PM208 (135.3 cM) on LG5. A total of 42.9% of phenotypic variation explained (PVE) was accounted for (LOD_s_ = 8.72) on LG1, followed by 14.5% (LOD_s_ = 3.57) on LG8A and 9.4% (LOD_p_ = 2.92) on LG5.

In the drought treatment population, large numbers of QTL clustered on LG1, located in the same region as the irrigated population between loci with linked markers MM135 and PM312 (CI: 51.8 cM–54.1 cM). The observation of overlapping confidence intervals with significant QTL controlling several traits in both irrigated and drought treatment population indicates the potential of intrinsic trait differences between parental genotypes which then segregate in the offspring population. Linkage group LG1 with confidence intervals between 51.8 cM and 54.1 cM were mapped with QTL for internode length (IR (Irrigated): 42.9%; LOD_s_ = 8.72; D (Drought): 40.2%; LOD_s_ = 9.06), peduncle length (IR: 41.1%; LOD_s_ = 8.93; D: 54.7%; LOD_s_ = 10.83), pod weight per plant (IR: 18.9%; LOD_s_ = 4.57; D: 22.8%; LOD_s_ = 4.47) and seed number per plant (IR: 28.1%; LOD_s_ = 6.47; D: 23.7%; LOD_s_ = 3.46) and were consistent across the irrigated and the drought treatment population. In addition, seed weight per plant (IR: 13.6%; LOD_s_ = 3.39; D: 19.3%; LOD_s_ = 3.16) also overlapped at a location between 84.9 cM and 91.4 cM on LG2.

The effect of the drought treatment influencing QTL in the drought treatment population was identified for some of the traits. Significant QTL for pod number per plant (33.9%; LOD_s_ = 7.01) and harvest index (24.5%; LOD_s_ = 5.43) were mapped in an overlapping confidence interval between 51.8 cM and 54.1 cM on LG1 in the irrigated population; however, these had been reduced to putative QTL with reduced phenotypic variation explained (18.2%; LOD_p_ = 2.57) and 16.1% (LOD_p_ = 2.74) in the drought treatment population, for the respective traits. Further drought treatment modified QTL were observed for harvest index in which a significant QTL was mapped on LG2 at 55.3 cM (CI: 53.3 cM–63.6 cM; 17.5%; LOD_s_ = 3.00) in the drought treatment population while a putative, but separate, QTL was mapped at 91.4 cM (CI: 84.9 cM–91.4 cM; 11.1%; LOD_p_ = 2.73) on LG2 in the irrigated population. For traits including 100-seed weight and harvest index, mapping of significant QTL on different linkage groups and in different locations on the same linkage group in the drought treatment population compared to the irrigated population reflected a likely interaction between the drought treatment and the agronomic traits in the segregating population. For instance, significant QTL for 100-seed weight were mapped on LG2 at 84.0 cM and LG 4 at 16.5 cM in the drought treatment population, but not in the irrigated treatment, with PVE of 19.1% (LOD_s_ = 3.97) and 14.8% (LOD_s_ = 3.21), respectively.

### 3.4. Comparison of the Irrigated and Drought Treatment Population for QTL Associated with Drought

Five traits that have classically been used to evaluate the physiological effects of drought, including stomatal conductance, relative water content, stomatal density, leaf carbon (Delta C^13^) isotope analysis (CID) and leaf (Delta N^15^) isotope analysis (NID), were evaluated for QTL, based on the expression-based (GEM) genetic linkage map (Figure 3). Stomatal density and NID were identified to have significant QTL in the irrigated population. QTL controlling stomatal density were identified on two linkage groups in the irrigated population. Stomatal density mapped onto LG1 between 81.9 cM and 98.6 cM with significant QTL for the irrigated (27.8%; LOD_s_ = 4.78) and drought treatment population (20.6%; LOD_s_ = 3.65), suggesting that the QTL controlling stomatal density on LG1 could be derived from the intrinsic genotypic difference between the parental lines. Another significant QTL for stomatal density in the irrigated population was found to be linked to marker PM424 at 2.65 cM on LG10 with PVE of 15.3% (LOD_s_ = 2.76). For NID, one significant QTL was detected on LG1 at 51.8 cM and two putative QTL on LG4 and LG7 at 19.5 cM and 61.2 cM, respectively, in the irrigated population. The PVE for NID was reported to be 31% (LOD_s_ = 5.25) on LG1, followed by 14.7% (LOD_p_ = 2.87) and 9.3% (LOD_p_ = 2.13) on LG 4 and LG7, while no significant QTL was found in the drought treatment population. No significant QTL were detected for relative water content, stomatal conductance and CID in the irrigated population.

A total of six QTL (five significant and one putative) was mapped on LG1, LG2, LG4 and LG5 in the drought treatment population for drought assessment traits. Most of the QTL were identified on LG2 at different positions, including significant QTL for stomatal conductance (43.5 cM) and CID (91.4 cM), as well as a putative QTL for stomatal density (87.9 cM). Significant QTL for stomatal conductance accounted for 22.3% of PVE (LOD_s_ = 3.23). CID and stomatal density were controlled by multiple QTL across different linkage groups. The most significant QTL associated with CID was located on LG2 and had a PVE of 22% (LOD_s_ = 3.46), while another significant QTL was mapped on LG5 (37.9 cM) with a PVE of 18.4% (LOD_s_ = 3.12). Mapping of significant QTL for stomatal conductance and CID in the drought treatment population, but not in the irrigated population on LG2 and LG5, suggested that the drought response differs between the two parental genotypes for these traits and segregated in this F_5_ segregating population. For stomatal density, the most significant QTL located on LG1 (92.9 cM) accounted for 20.6% (LOD_s_ = 3.65), followed by a QTL on LG4 (6.6 cM) with PVE of 20.3% (LOD_s_ = 3.52). The putative QTL on LG2 only accounted for 11.5% (LOD_p_ = 2.54). No significant nor putative QTL were identified for relative water content and NID in the drought treatment population.

In addition to a graphical presentation of QTL location (Figure 3), a summary of QTL associated with 17 studied traits, LOD score, position of QTL, location of nearest markers, PVE and additive effect is also presented in Table 3.

## 4. Discussion

### 4.1. Novel GEMs Generated Using Affymetrix Soybean Genome GeneChip

The use of a microarray offers the potential to obtain both gene expression variation (pseudo-phenotypic data) and genotypic markers for the construction of a genetic linkage map, allowing the identification of thousands of markers in a single experiment. When integrated with trait QTL, the causal loci within the QTL genomic regions and the hypothetical regulatory networks controlling phenotypic variation could, potentially, be analysed. Despite the transcriptome sequence information for bambara groundnut not being fully available and the lack of a genome sequence, the development of a novel mapping marker system through cross-hybridising bambara groundnut RNA onto the Affymetrix Soybean Genome GeneChip allowed the construction of a high density expression-based genetic map for bambara groundnut with subsequent trait QTL analysis, as well as permitting a comparison of the genomic location of the detected genetic effects in bambara groundnut (QTL) with the equivalent genomic locations in soybean. An example of using cross-species approaches to translate genomic resources from model plants to a less intensively studied plants was reported in cowpea [18]. A total of 1058 single feature polymorphism (SFPs), which are microarray-based markers, were detected and validated in cowpea using Affymetrix Soybean Genome GeneChip, enabling subsequent high-throughput genotyping and high density mapping in cowpea [18].

In previous publications, the development of GEMs is based on the average hybridisation signal produced from a single probe-set, which is represented by ~11 probe-pairs when the Affymetrix GeneChip is used or by a number of features when an Agilent chip is used (usually 1–3 60-mer probes). The first approach in which the hybridisation signal was measured at the level of the soybean probe-sets identified 15 (0.02%) potential GEMs out of 61,035 probe-sets, and this may be due to the hybridisation of RNA samples from bambara groundnut onto a heterologous soybean genome microarray (evolutionary separation of the two species being around 20 million years; [27]). Despite reasonably high signal strength that might be generated by one probe-pair in a probe-set, the hybridisation signal is averaged across all probe-pairs that represent that probe-set. Poor hybridisation to other probe-pairs could reduce the overall mean, and as a result, a relatively low distinctness score could be produced, resulting in few GEMs being selected. The results improved when probe-sets were masked with a custom made CDF file to remove probe-pairs with poor hybridisation signal. Forty-eight genes out of 53,651 (0.09%) were selected as GEMs.

However, the number of GEMs generated at probe-set (15) and CDF masked probe-set level (48) was insufficient for use in genetic linkage analysis as a single marker type. The development of GEMs at the probe-pair level was established to overcome the likely signal damping effect resulting from averaging of the signal across all probe-pairs in each probe-set. From the list of probe-pairs with different distinctness scores, differential hybridisation to the probe-pairs from the same probe set was also discovered, for example: Gma.12360.1.S1_at; PM-394638, Gma.12360.1.S1_at; PM-1346833 and Gma.12360.1.S1_at; PM-432117, which showed distinctness scores of 3.72, 2.08 and 1.73, respectively. The analysis of the hybridisation signal data at the probe-pair level offers an advantage in terms of retrieving potential probe-pairs with a high distinctness score and to remove poorly-hybridised probe-pairs from each probe-set in order to obtain as much information as possible for GEMs’ development.

Of 669,982 probes, 1029 PM probes and 501 MM probes (0.23%) were chosen as potential GEMs when the analysis was conducted at the probe-pair level. A similar number of GEMs were used for map construction (838 putative GEMs out of 92 k transcripts from the Agilent Brassica 60-mer array; [14]). The current cross-species analysis has not only to contend with the small number of genes actually showing a DNA-based (sequence) or RNA-based (expression level) difference during a homologous species-chip analysis, but must also contend with lower signal strength due to evolutionary distance between target species and the microarray used.

A distinctness score was used to enrich for the separation of ‘a’ and ‘b’ allele scores across the individual lines, allowing the probe-pairs to be selected as potential GEMs. A high distinctness score should distinguish between ‘a’ and ‘b’ allele across individual lines and assign them, largely, into two distinct groups. For example, Gma.15877.1.S1_at; PM-41730, with a distinctness score of 5.31 produced two distinct groups that showed hybridisation signal as high as 2360.75 (‘a’ allele, average: 1938.53; s.d.: 218.97) and as low as 256.44, respectively (‘b’ allele, average: 395.16; s.d.: 80.63). The most likely explanation of such large differences would be the accidental sequence homology of a repetitive element present in one genotype, but not the other. For the associated GEM to map would require that the repetitive sequence were randomly repeated at only a single locus. For decreased values of the distinctness score, the distribution of the two allele groups becomes more scattered, and there is the potential of having a hybridisation signal from some individual lines falling in between the two distinct groups, for instance GmaAffx.71175.1.S1_s_at; PM-1195578 with a distinctness score of 3.82. This noise could be due to technical issues, such as the strength of the hybridisation of nucleic acids onto an individual GeneChip (although all chips were normalised before analysis) and risks misclassification of allele types. Thus, a series of cut-off points are set during data analysis in order to remove probe-pairs with poor performance, very similar signal or with high scatter across lines. It is also worth noting that the current cross is at the F_5_ generation, so 1/16th heterozygosity would be predicted to remain in the lines, per marker, on average (6.25%), and 3–4 individuals in the population might be expected to be heterozygous for any marker. If the markers behave in a co-dominant (dosage dependent) way, then there are likely to be a number of intermediate a/b markers, which would reduce the distinctness score and could lead to marker rejection.

### 4.2. Use of GEMs for Genetic Mapping

In terms of quantity, the use of both PM probes and MM probes increases the number of markers available for genetic mapping. The spaced framework genetic map, which was produced after repetitive rounds of removing markers, is expected to represent the most robust markers with reasonable overall coverage for the F_5_ segregating population of 65 individual lines in bambara groundnut. The PM probes and MM probes in each probe-pair could have different hybridisation signals due to the single nucleotide difference present by the design at the 13th nucleotide between the PM and MM probe of a probe-pair. This could result in a variation of the distinctness score and might give some indication of the basis of the polymorphism mapped.

The first priority in map construction must be to use the best quality data to produce the most accurate map, then additional putative markers could be introduced using less stringent criteria, but fixing the order of the framework map first. If the data quality is good, additional markers will allow denser maps to be constructed. If the additional marker quality is poor, approximate positions can still be assigned, which could be useful in any conserved synteny comparisons or subsequent fine mapping.

As GEMs are potentially ‘transient’ markers, the integration of GEMs into a stable DNA sequence-based framework map is recommended [12]. There are several potential advantages for the integration of maps. Integration of maps allows the potential alignment of GEMs and thus also the DNA sequence-based framework map to the soybean physical map. A detailed integrated map in bambara groundnut would be necessary in future to offer greater potential to map QTL with traits of interest more accurately.

### 4.3. Association between Markers and Traits in Bambara Groundnut

A similar pattern of QTL controlling the studied common traits in both irrigated and drought treatment populations suggested that most of the detected QTL are the result of intrinsic differences between the parental lines used to create the segregating population, with limited responses to the imposed drought. Several QTL were found to effect internode length and peduncle length in both drought and irrigated populations with the significant QTL consistently mapped to LG1 in the segregating population across the two treatments. However, there were minor QTL effecting internode length, such as QTL in LG8A, which explained 14.5% (LOD_s_ = 3.57) and 9.1% (LOD_p_ =2.59) in the irrigated population and drought treatment population, respectively. The inheritance of internode length was contributed to by one major QTL plus a few minor QTL. The major QTL linked with the same marker on LG1 suggested that these two traits are probably largely controlled by a single gene or two closely-linked genes. The hypothesis may be supported by Basu et al. (2007) [8] who reported that the segregation pattern of internode length was consistent with primarily monogenic inheritance in a domesticated (DipC) line crossed with a *V. subterranea* var. *spontanea* (VSSP11), created to evaluate the domestication process in bambara groundnut. This detected locus could represent residual variation at the same gene whose domestication led to the bunchy morphology.

The present study showed the potential interaction of complex traits, such as pod number per plant and 100-seed weight, with environmental factors (such as drought) and underlying the genes controlling yield traits, given that they are downstream traits. The discovery of a number of QTL (rather than a single major locus) explaining more limited phenotypic variation for yield traits suggested that these traits could probably be controlled by many genes with minor effects, as has been generally reported for yield. For instance, 100-seed weight was contributed to by multiple loci located across LG2, LG4 and LG11, explaining 19.1%, 14.8% and 12.8% of the phenotypic variation, respectively, in the drought treatment population. The observation of multiple genes with minor effects controlling yield-related traits was also reported by Zhang et al. (2004) [28] who discovered four QTL located on three linkage groups (A2, B1 and D2) for seed weight in RILs derived from soybean vars. *Kefeng No.1* X *Nannong 1138-2*.

The present study also showed that QTL controlling internode length, peduncle length, pod number per plant and seed number per plant were linked with the same marker MM135 at 51.8 cM on LG1. The clustered QTL on LG1 could correspond to a single gene controlling plant architecture, which has pleiotropic effects on different traits, including seed and plant growth-related traits. In pea, QTL detected for seed traits were found to be located in the genomic regions regulating traits, such as plant morphology, phenology and plant biomass [22]. The authors showed that the *Le* allele, which is related to internode length, has pleiotropic effects on other traits, such as plant height, vegetative biomass and plant nitrogen content. In wheat, the dwarfism gene (*Rht-1*) is associated with many QTL, including grain yield and root development QTL [29]. In rice, a single gene controlling erect leaf development is associated with higher grain yield [30].

For drought-related traits, QTL for stomatal conductance, carbon isotope discrimination analysis and stomatal density were largely located on LG2 in the drought treatment population. Mild drought treatment was expected to activate gene expression in bambara groundnut in response to stress, leading to identification of response-associated QTL, specifically in the drought treatment population only. The location of QTL for NID was detected in irrigated plants on LG1 (51.8 cM; CI: 47.5 cM–54.1 cM), corresponding to loci that linked with internode length, pod number per plant, pod weight per plant and seed number per plant (CI: 47.5 cM–54.1 cM). The co-located QTL illustrate the positive relationship between nitrogen assimilation and biomass in plants. Coque et al. (2006) [31] reported that twelve QTL mapped with ^15^N abundance were involved in nitrogen use efficiency, such as grain yield, N uptake and N remobilisation. However, the non-significant QTL for NID in drought treatment plants could be explained by the effect of mild drought imposed on the plants and potential limitations to uptake of N (given that it is water soluble) and reduced biomass production, resulting in a relatively weak association between loci and traits.

The application of QTL analysis can be extended to the identification of candidate genes that control these respective traits. An example of using cross-species approaches to identify candidate genes is reported in cowpea. Through the syntenic relationship between cowpea with *Medicago truncatula* and soybean, the syntenic locus for *Hls* (hastate leaf shape) was discovered and led to the identification of a candidate gene controlling leaf morphology in cowpea [32]. The cross-species approach presented in cowpea provides an alternative option to identify candidate genes in underutilised crop species.

## 5. Conclusions

This study produced GEMs at the (unmasked) probe-pair (oligonucleotide) level after cross-hybridising leaf RNA from a segregating bambara groundnut cross under a mild drought treatment with the Affymetrix Soybean Genome GeneChip. This is the first development of GEMs in bambara groundnut, and they are expected to represent either differences in the hybridisation signal of RNA to individual oligonucleotide probes or genuine differences in gene expression levels. A first spaced GEM map was then developed, and this is also the first ‘expression-based’ map in bambara groundnut. The first comprehensive QTL analysis with good genome coverage using the GEM map showed the usefulness of the GEM map in mapping both intrinsic and drought-related QTL in the F_5_ segregating population. This study also serves as a starting point for fine mapping, which allows targeted QTL identification for further application in marker-assisted selection within this minor legume crop, which already has good drought tolerance characteristics. That QTL were discovered relating to stomatal conductance, carbon isotope discrimination analysis and stomatal density of the drought assessment traits suggests that the two parental lines do differ in their response to drought, and a more detailed analysis may allow these mechanisms to be elucidated.

## Figures and Tables

**Table 1:** A summary of gene expression markers (GEMs) development at three different levels: probe-sets, chip definition file (CDF) masked probe-sets and unmasked oligonucleotides.

Level of Analysis	Highest Distinctness Score	Cut-Off Point	Total Potential GEMs	Number of Actual GEMs
Probe-sets	4.03	2.50	61,035	15
CDF masked probe-sets	6.09	2.60	53,651	48
Unmasked probe-pairs (Oligonucleotides)				
(a) Perfect-match probes (PM)	7.99	2.34	669,982	1029
(b) Mismatch probes (MM)	7.10	2.30	669,982	501

**Table 2:** The distribution of gene expression markers (GEMs) across 13 LGs for genetic linkage analysis in the F_5_ segregating population of bambara groundnut.

Linkage Group (LG)	Length of LG (cM)	GEMs	Average Marker Interval (cM)
1	114.4	20	5.7
2	91.4	17	5.4
3	94.0	20	4.7
4	47.9	12	4.0
5	135.3	27	5.0
6A	68.0	14	4.9
6B	16.2	8	2.0
7	73.9	16	4.6
8A	60.1	17	3.5
8B	7.5	6	1.3
9	73.8	18	4.1
10	95.2	23	4.1
11	105.0	20	5.3
Grand total	982.7	218	54.6
Mean	75.6	16.8	4.2

**Table 3:** The identification of QTL controlling 17 traits involved in agronomy and drought responses in both irrigated and drought treatment F_5_ segregating population.

* Traits	Treatment	QTL- LG	Position (cM)	Nearest Marker	Marker Interval	LOD	PT	PVE%	Additive Effect
DE	-	11	15.1	PM289 (15.1 cM)	MM152–PM269	2.12 ^p^	2.80	15.3	0.05
DF	-	8B	7.5	PM271 (7.5 cM)	PM267	3.79	2.80	25.6	1.32
EDP	IR	11	101.3	MM130 (99.3 cM)	MM130–MM161	1.84 ^ns^	3.00	13.4	1.27
	D	5	112.8	PM177 (117.8 cM)	PM260–PM177	2.64 ^p^	3.00	18.6	−1.78
IN	IR	1	52.8	MM135 (51.8 cM)	MM135–PM312	8.72	3.10	42.9	−0.64
		8A	55.1	PM341 (55.6 cM)	PM395–PM341	3.57	3.10	14.5	−0.36
		5	134.8	PM208 (135.3 cM)	PM112–PM208	2.92 ^p^	3.10	9.4	0.30
	D	1	52.8	MM135 (51.8 cM)	PM135–PM312	9.06	3.00	40.2	−0.69
		11	55.6	PM115 (52.6 cM)	PM115–MM149	2.85 ^p^	3.00	9.8	0.35
		8A	60.1	PM428 (60.1 cM)	PM341	2.59 ^p^	3.00	9.1	−0.33
PEL	IR	1	53.8	PM312(54.1 cM)	MM135–PM312	8.93	3.10	41.1	−1.01
		2	4.5	PM187 (4.8 cM)	PM486–PM187	2.87 ^p^	3.10	10.4	0.51
		7	73.9	MM220 (73.9 cM)	PM166	2.21 ^p^	3.10	6.5	−0.43
	D	1	52.8	MM135 (51.8 cM)	PM135–PM312	10.83	3.00	54.7	−1.21
		11	56.6	PM115 (52.6 cM)	PM115–MM149	2.32 ^p^	3.00	8.4	0.49
PN	IR	1	52.8	MM135 (51.8 cM)	MM135–PM312	7.01	3.00	33.9	−15.21
		11	60.3	MM178 (60.3 cM)	MM149–PM301	3.15	3.00	13.5	9.29
		5	132.8	PM112 (132.8 cM)	PM23–PM208	2.64 ^p^	3.00	11.1	8.52
	D	1	51.8	MM135 (51.8 cM)	PM45–PM312	2.57 ^p^	3.00	18.2	−11.69
PW	IR	1	53.8	MM135 (51.8 cM)	MM135–PM312	4.57	3.10	18.9	−10.60
		11	60.3	MM178 (60.3 cM)	MM149–PM301	3.57	3.10	15.1	9.33
		2	91.4	MM232 (91.4 cM)	PM340	2.56 ^p^	3.10	10.4	7.83
	D	1	52.8	MM135 (51.8 cM)	PM135–PM312	4.47	3.00	22.8	−10.26
		11	56.6	MM149 (59.2 cM)	PM115–MM149	3.61	3.00	17.5	9.30
		2	58.3	PM190 (53.3 cM)	PM190–PM84	2.49 ^p^	3.00	11.1	9.55
SN	IR	1	52.8	MM135 (51.8 cM)	MM135–PM312	6.47	3.00	28.1	−15.14
		11	60.3	MM178 (60.3 cM)	MM149–PM301	3.82	3.00	15.7	11.06
		2	60.3	PM84 (63.6 cM)	PM190–PM84	3.2	3.00	12.4	13.04
	D	1	51.8	MM135 (51.8 cM)	PM45–PM312	3.46	3.00	23.7	−14.04
SW	IR	11	60.3	MM178 (60.3 cM)	MM149–PM301	3.99	3.10	16.5	6.97
		2	91.4	MM232 (91.4 cM)	PM340	3.39	3.10	13.6	6.41
		1	54.1	PM312(54.1 cM)	PM312–PM261	3.25	3.10	13.4	−6.53
	D	2	91.4	MM232 (91.4 cM)	PM340	3.16	3.00	19.3	6.63
		1	51.8	MM135 (51.8 cM)	PM45–PM312	2.45 ^p^	3.00	12	−5.39
		11	55.6	PM115 (52.6 cM)	PM115–MM149	2.17 ^p^	3.00	12.5	5.76
100-SW	IR	9	52.4	PM181 (55.4 cM)	MM238–PM181	4.49	3.00	27.5	−7.62
		10	21.6	MM131 (22.4 cM)	PM98–MM131	2.21 ^p^	3.00	12.7	4.84
		11	30.2	PM204 (30.2 cM)	PM393–MM37	2.38 ^p^	3.00	11.4	4.53
	D	2	84.0	PM247 (84.0 cM)	PM209–PM340	3.97	3.00	19.1	5.53
		4	16.5	MM25 (12.5 cM)	MM25–PM164	3.21	3.00	14.8	−5.52
		11	28.7	PM204 (30.2 cM)	PM393–PM204	2.8 ^p^	3.00	12.8	4.62
SDW	IR	11	60.2	MM178 (60.3 cM)	MM149–MM178	5.62	3.00	27.7	9.73
		8A	56.6	PM341 (55.6 cM)	PM341–PM428	2.67 ^p^	3.00	11.3	−6.29
		1	61.0	PM261 (58.0 cM)	PM261–PM388	2.28 ^p^	3.00	9.7	−6.18
	D	1	51.8	MM135 (51.8 cM)	PM45–PM312	3.9	2.90	22.4	−8.63
		11	55.6	PM115 (52.6 cM)	PM115–MM149	3.67	2.90	20.2	8.81
HI	IR	1	54.1	PM312 (54.1 cM)	MM135–PM261	5.43	3.00	24.5	−0.16
		2	91.4	MM232 (91.4 cM)	PM340	2.73 ^p^	3.00	11.1	0.11
	D	2	55.3	PM190 (53.3 cM)	PM190–PM84	3	3.00	17.5	0.12
		1	52.8	MM135 (51.8 cM)	PM135–PM312	2.74 ^p^	3.00	16.1	−0.10
		11	27.7	PM204 (30.2 cM)	PM393–PM204	2.2 ^p^	2.80	10.6	0.09
RWC	IR	10	2.1	PM136 (2.1 cM)	MM197–PM424	1.86 ^ns^	2.90	13.5	0.58
	D	5	0.0	MM89 (0.0 cM)	MM82	1.35 ^ns^	3.00	10	0.38
SC	IR	3	24.7	PM103 (24.7cM)	MM56–MM235	1.17 ^ns^	2.60	8.7	15.83
	D	2	43.5	MM61 (42.5 cM)	MM61–PM190	3.23	3.00	22.3	17.76
CID	IR	3	8.9	PM327 (4.6 cM)	PM327–PM211	1.63 ^ns^	3.00	12.5	0.46
	D	5	37.9	MM54 (54.7 cM)	MM179–MM54	3.23	3.00	18.4	−0.60
		2	91.4	MM232 (91.4 cM)	PM340	3.46	3.00	22	−0.43
SD	IR	1	87.9	PM96 (81.9 cM)	PM96–PM213	4.78	2.50	27.3	2.25
		10	2.7	PM424 (2.7 cM)	PM136–PM191	2.76	2.50	15.3	−1.41
	D	1	92.9	PM213 (98.6 cM)	PM96–PM213	3.65	3.00	20.6	1.43
		4	6.6	PM372 (6.6 cM)	PM315–PM422	3.52	3.00	20.3	1.23
		2	87.9	MM232 (91.4 cM)	PM340–MM232	2.54 ^p^	3.00	11.5	−0.98
NID	IR	1	51.8	MM135 (51.8 cM)	PM45–PM312	5.25	3.00	31	−0.46
		4	19.5	MM25 (12.5 cM)	MM25–PM164	2.87 ^p^	3.00	14.7	0.39
		7	61.2	PM378 (61.7 cM)	PM298–PM378	2.13 ^p^	3.00	9.3	−0.27
	D	11	51.1	PM83 (51.1 cM)	PM389–PM52	1.64 ^ns^	3.00	12.4	−0.28

ns: non-significance at *p* ≤ 0.05 by the permutation test using 10,000 reiterations. p: putative QTL where the LOD score is lower than the GW threshold by up to a 1-LOD interval. PT: permutation test threshold using 10,000 reiterations at *p* ≤ 0.05. * DE, days to emergence; DF, days to flowering; EDP, estimated days to podding; IN, internode length; PEL, peduncle length; PN, pod number per plant; PW, pod weight per plant; SN, seed number per plant; SW, seed weight per plant; 100-SW, 100-seed weight; SDW: shoot dry weight; HI, harvest index; SC, stomatal conductance; CID, leaf carbon (Delta C^13^) isotope analysis; SD, stomatal density; NID, leaf (Delta N^15^) isotope analysis.

## Supplementary Materials

The following are available online at www.mdpi.com/2073-4425/8/2/84/s1: Supplemental Text S1: Generations of GEMs; Table S1: Pearson’s Correlation Coefficients between agronomic traits measured in both irrigated and drought treatment F_5_ segregating population.
