# Supplementary material for: A Cross-Species Gene Expression Marker-Based Genetic Map and QTL Analysis in Bambara Groundnut
Source: Genes (Basel). 2017 Feb 22;8(2):84. doi: 10.3390/genes8020084 (PMC5333073; doi:10.3390/genes8020084)
Supplement: Supplementary file 1 [file genes-08-00084-s001.docx]

Supplementary Materials: A Cross-Species Gene Expression Marker-Based Genetic Map and QTL Analysis in Bambara Groundnut

Hui Hui Chai, Wai Kuan Ho, Neil Graham, Sean May, Festo Massawe and Sean Mayes

Supplemental Text S1: Generation of GEMs

A series of analyses were conducted to generate potential gene expression markers (GEMs). Firstly, the mean and standard deviation (s.d.) of each log2-normalised hybridisation signal were calculated for each of the parents from the drought plot (Tiga Nicuru (*n* = 3) and DipC (*n* = 3)), followed by the segregating population (*n* = 65) for each putative marker. Secondly, each individual line for each putative marker was provisionally assigned into parental “Tiga Nicuru” and “DipC” scores based on the mean of the signal value of the population (*n* = 65). Conventionally, the female parent is represented as the first parent in a cross. Here, the female parent is Tiga Nicuru and the male parent DipC. An “a” allele score was given when the signal value of an individual line was on the same side of the mean population signal as the Tiga Nicuru parent. A “b” score was given when the hybridisation signal for an individual line was on the same side of the mean as the parental value “DipC”. Thirdly, the mean and s.d. of the signal value were computed for individual lines scored as “a” and “b”, respectively. The s.d. values from “a” and “b” for each marker were averaged. By dividing the s.d. of the hybridisation signal of the entire population by the average s.d. of the hybridisation signal derived from “a” and “b”, a “distinctness” score that indicated the likely degree of separation between group “a” and group “b” was calculated. The probe-sets or probe-pairs with distinctness score of equal or higher than a selected threshold value were selected as potential GEMs.


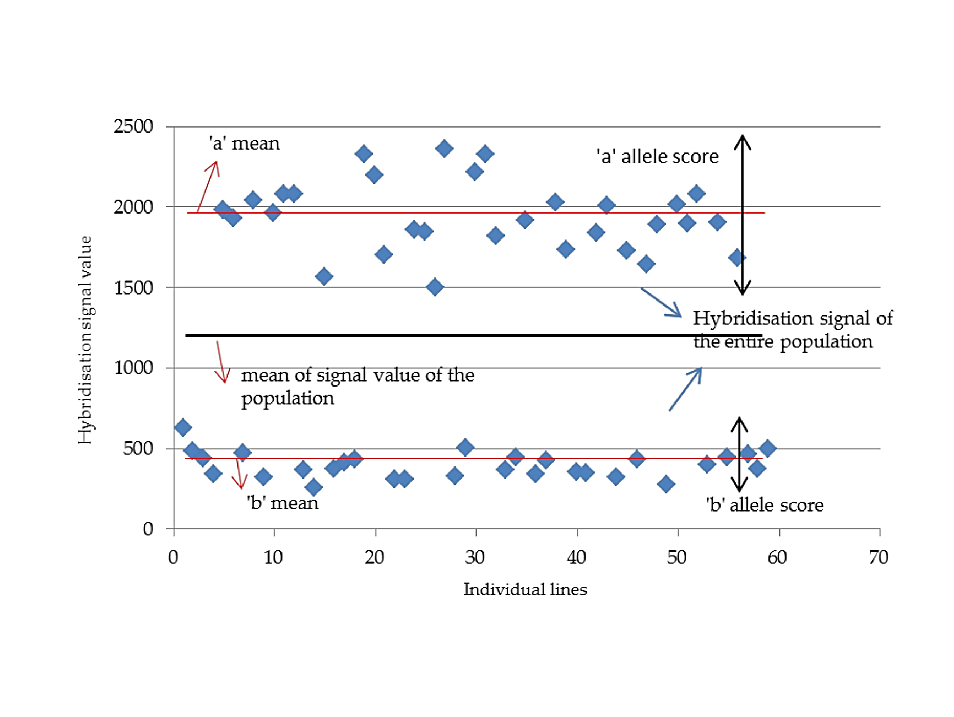


**Figure S1.** An illustration of the estimates generated to develop the ”distinctness” score for
potential GEMs.

**Table S1.** Pearson”s correlation coefficients between agronomic traits measured in both irrigated and drought treatment F_5_ segregating populations.

| Estimated days to podding_IR | 1 | - |  |  |  |  |  |  |  |  |  |
| --- | --- | --- | --- | --- | --- | --- | --- | --- | --- | --- | --- |
| Estimated Days to podding_D |  | - |  |  |  |  |  |  |  |  |  |
| Internode length_IR | 2 | 0.03 | - |  |  |  |  |  |  |  |  |
| Internode length_D |  | 0.11 | - |  |  |  |  |  |  |  |  |
| Peduncle length_IR | 3 | −0.03 | 0.82 ** | - |  |  |  |  |  |  |  |
| Peduncle length_D |  | 0.21 | 0.80 ** | - |  |  |  |  |  |  |  |
| Shoot dry weight_IR | 4 | −0.06 | 0.63 ** | 0.55 ** | - |  |  |  |  |  |  |
| Shoot dry weight_D |  | 0.12 | 0.60 ** | 0.59 ** | - |  |  |  |  |  |  |
| Pod number per plant_IR | 5 | 0.01 | 0.67 ** | 0.58 ** | 0.79 ** | - |  |  |  |  |  |
| Pod number per plant _D |  | 0.10 | 0.54 ** | 0.47 ** | 0.88 ** | - |  |  |  |  |  |
| Pod weight per plant_IR | 6 | −0.09 | 0.67 ** | 0.63 ** | 0.86 ** | 0.93 ** | - |  |  |  |  |
| Pod weight per plant_D |  | −0.01 | 0.61 ** | 0.58 ** | 0.89 ** | 0.87 ** | - |  |  |  |  |
| Seed number per plant_IR | 7 | 0.01 | 0.66 ** | 0.59 ** | 0.79 ** | 0.98 ** | 0.94 ** | - |  |  |  |
| Seed number per plant _D |  | 0.12 | 0.59 ** | 0.53 ** | 0.87 ** | 0.98 ** | 0.89 ** | - |  |  |  |
| Seed weight per plant_IR | 8 | −0.11 | 0.60 ** | 0.58 ** | 0.85 ** | 0.89 ** | 0.99 ** | 0.93 ** | - |  |  |
| Seed weight per plant_D |  | −0.06 | 0.56 ** | 0.53 ** | 0.85 ** | 0.85 ** | 0.99 ** | 0.87 ** | - |  |  |
| 100-seed weight_IR | 9 | −0.28 * | 0.18 | 0.29 * | 0.48 ** | 0.22 | 0.49 ** | 0.25 * | 0.54 ** | - |  |
| 100-seed weight_D |  | −0.42 ** | 0.17 | 0.21 | 0.23 | 0.11 | 0.43 ** | 0.07 | 0.49 ** | - |  |
| Harvest index_IR | 10 | −0.10 | 0.59 ** | 0.61 ** | 0.53 ** | 0.81** | 0.85 ** | 0.83 ** | 0.84 ** | 0.42 ** | - |
| Harvest index_D |  | −0.18 | 0.50 ** | 0.48 ** | 0.60 ** | 0.67** | 0.86 ** | 0.71 ** | 0.89 ** | 0.61 ** | - |
|  |  | 1 | 2 | 3 | 4 | 5 | 6 | 7 | 8 | 9 | 10 |

* Significant level of *p* < 0.05; ** Significant level of *p* < 0.01. IR, Irrigated; D, Drought.
